# The anti-tumor effect and increased tregs infiltration mediated by rAAV-SLC vector

**DOI:** 10.1007/s11033-013-2663-7

**Published:** 2013-09-28

**Authors:** Rilun Li, Heng Hu, Huiying Ma, Long Chen, Shuang Zhou, Binbin Liu, Yinkun Liu, Chunmin Liang

**Affiliations:** 1 Department of Anatomy and Histology & Embryology, Shanghai Medical College of Fudan University, 138 Yixueyuan Road, Shanghai, 200032 People’s Republic of China; 2 Liver Cancer Institute of Zhongshan Hospital, Fudan University, Shanghai, 200032 People’s Republic of China

**Keywords:** Recombinant adeno-associated virus, Secondary lymphoid tissue chemokine, Anti-tumor effect, Tregs

## Abstract

To explore the anti-tumor effect and immune mechanism mediated by a new recombinant adeno-associated virus (rAAV) encoding secondary lymphoid tissue chemokine (SLC) mature peptide gene. AAV Helper-Free system was used for rAAV-SLC package. The anti-tumor effect of SLC was detected by bearing tumor established from Hepal-6 cells both in C57BL/6J and nude mice. Flow cytometry analysis and IHC for Tumor-infiltrating T cells and CD11c+DCs were also investigated to explore the immunological mechanism. rAAV-SLC was successfully packaged in AAV293 cells and transfected Hepal-6 tumor cells at high efficiency. The anti-tumor effect was demonstrated by less tumor weight and longer survival outcome. Coincident with the anti-tumor response, local elaboration of SLC within the tumor bed elicited a heavy infiltration of CD4^+^, CD8^+^T cells and CD11c^+^ dendritic cells into the tumor sites. More importantly, there was higher infiltration of Foxp3+ regulatory T cells (Tregs). Local elaboration of SLC mediated by rAAV-SLC has strong T cell mediated anti-tumor effect. The study also suggested that Tregs in the tumor microenvironment tampered the anti-tumor effect.

## Introduction

Chemokines are small diffusible or matrix-associated proteins that transmit chemotactic signals into cells through pertussis toxin-sensitive, G protein-coupled receptors. Among those chemokines, the chemokine secondary lymphoid tissue chemokine (SLC/CCL21) has been demonstrated to be unique one. It has been believed SLC and its receptor CCR7 play an important role in establishing the functional micro-environment in the secondary lymphoid tissues [1–12]. SLC is constitutively produced by high endothelial venules and by stromal cells within T cell zones of lymphoid organs. CCR7 is present on T cell subpopulations and is up-regulated by maturing dendritic cells and helps direct the immune effector cells to secondary lymphoid organs [6, 13, 14]. A spontaneous mouse line lacking SLC/CCL21 [15] and CCR7 knockout mice [16] show impaired homing of T cells into lymph nodes and Peyer’s patches within the small intestine. In addition to CCR7, SLC also interacts with the CXCR3 receptor, through which block angiogenesis in vivo [17]. Previous work demonstrated that the chemotactic activity of SLC for DCs and T cells could be harnessed to generate antitumor immune responses [18–20]. Intratumoral injection of SLC-gene-modified DCs resulted in more significant tumor growth inhibition [21]. It can be estimated that stronger response will be induced by attracting much larger numbers of effector T cells and APCs to the site of tumor, if a more efficient expression system established to produce SLC in tumor bed. The efficiency of an expression vector is one of the key aspects for gene therapy. Recently, it has been extensively demonstrated that recombinant adeno-associated virus (rAAV) are emerging nonpathogenic vectors with potential for cancer gene therapy. rAAV can successfully infect and transduce a broad variety of cell and tissue types, such as brain, liver, muscle, etc. [22–25]. Over the last several years, some in vivo studies using AAV have shown efficacious results in the treatment of multiple diseases in animal models and in human clinical trials [26, 27].

In this study, we evaluated the potential of rAAV as a vector for in vivo SLC gene modification of tumors and tested its antitumor properties.

## Materials and methods

### Animals

C57BL/6J (H-2^b^), BALB/c (H-2^d^) and female nude mice, all 6–8 weeks of age, were purchased from Chinese Academy of Sciences and housed at the Animal Maintenance Facility of the Fudan University Shanghai Medical College.

### Medium and chemokine

Complete medium consisted of RPMI 1640 with 10 % heat-inactivated FCS, 0.1 mM nonessential amino acids, 1 μM sodium pyruvate, 2 mM fresh l-glutamine, 100 μg/ml streptomycin, 100 units/ml penicillin, 50 μg/ml gentamicin, 0.5 μg/ml fungizone. Serum-free Opti-MEM I medium (GIBCO-BRL, Gaithersburg, Md.) Recombinant murine SLC was obtained from BIODESIGN (Saco,ME); Rat-anti-mouse SLC antibody was purchased from eBioScience company.

### Cell lines

A mouse hepatocellular carcinoma cell line, Hepal-6 tumor cells (ATCC, catalog No: CRL-1830) were purchased from Chinese Academy of Sciences. AAV293 cells were purchased from Stratagen System Company.

### Production and purification of rAAV-SLC

AAV Helper-Free system (Stratagene System Company, USA) was used for rAAV package. To amplify the fragments containing full coding region, the following specific primers of murine chemokine SLC was used. 5′AGC GAA TTC TAC AGC TCT GGT CTC ATC CTC A 3′ (sense), cloned into EcoR | site; 5′GCG CTC GAG GTC TCT TTT CTA GCT CCC TCT TTG 3′ (antisense), cloned into Xho | site. Total cellular RNA was extracted from lymph nodes from C57BL/6 J mice. The above fragments were amplified by RT-PCR (QIAGEN, Germany). After confirming the entire nucleotide sequence of PCR products, the EcoRI-XhoI fragment was inserted into the multiple cloning sites (MCS) of the pAAV-IRES-hrGFP vector, which contains the CMV promoter and AAV2 inverted terminal repeats (ITRs). rAAV-SLC serotype 2 vectors were prepared by using the three-plasmid cotransfection system. In addition, hrGFP expression was used to ascertain the transduction efficiency of the respective target. AAV293 cells were harvested 72 h post-transfection and lysed by repeated freezing and thawing. DNase|treatment of viral preparations was performed to digest unencapsidated DNA, and Real-time Quantitative PCR (QIAGEN) analysis was used to determine the particle genome titer.

### Cy3-labeled viral transduction

To investigate the infectious entry pathway of rAAV-SLC and assess AAV-mediated gene transfer, purified virus was labeled with the carbocyanine dye Cy3 (Amersham, NJ, USA). Hepal-6 cells were infected with 1 × 10^11^ particles/ml of Cy3-conjugated rAAV (~10^6^ particles/cell) in binding buffer (DMEM containing 2 mM glucose, 10 mM HEPES [pH 7.3], and 1 % bovine serum albumin) at 37 °C unless otherwise noted. Cells were washed 3 times with binding buffer prior to infection. Prior to fixation, the binding buffer was removed and the cells were washed 3 times again. Cells were then fixed with 4 % paraformaldehyde in PBS for 15 min at room temperature and washed 3 times with PBS. Where indicated, cells were either treated for 5 min at room temperature with 1-μg/ml DAPI (Inc., Eugene, OR) in PBS with 0.1 % Triton X-100 and washed 3 times with PBS or mounted in medium containing DAPI, which was used to indicate the position of the cell nucleus. Distribution of Cy3-AAV particles in Hepal-6 cells at different time points (Control, 60 min, 2 h, 12 h, 24 h, 72 h) post infection was analyzed by confocal microscopy.

Twenty-four hours before transfection, Hepal-6 cells were plated in growth medium without antibiotics and grew to be approximately 80 % confluent. The cells were washed once with serum free Opti-MEM I medium, and incubated with 100 MOI of rAAV for different time points (control, 60 min, 2 h, 12 h, 24 h, 72 h) at 37 °C with 7 % CO2 with gentle agitation every 15 min. After infection, cells were washed three times and then cultured in complete RPMI medium until further analyses.

### Detection of SLC produced by Hepal-6 cells infected with rAAV-SLC

SLC expression of Hepal-6 cells post-transfection was confirmed by RT-PCR and specific ELISA. Supernatants were collected from Hepal-6 cells with either rAAV-GFP or rAAV-SLC 48 h post-infection and stored in −76 °C for further use. A chemotaxis assay was used to further determine the levels and the bioactivity of SLC produced in the supernatant of SLC-modified Hepal-6 cells. Supernatants from infected cells were added to the bottom chamber of 24-well plates with 6.5-mm diameter, 5 μm pore polycarbonate Transwell insets (Corning Coster, Cambridge, MA) in duplicate samples, as the source of chemo attractant in chemotaxis assays, with T cells (10^7^) being performed in the upper chamber as responder cells. At the same time, recombinant mouse SLC (100 ng/ml, BIODESIGN, ME) were added to the bottom chamber as positive control. T cells were purified from normal C57BL/6J mouse spleen using mouse T cell recovery column kit (Cedarlane, Ontario, Canada) according to the instructions of the manufacturer. For determining the number of cells, 10^4^ polystyrene beads (15-μm diameter; Bangs Laboratories, Fishers, IN) were added to the lower chamber previously. Samples were stained with antibodies against CD3 (for T cells) and counted on a FACS caliber (Becton–Dickinson, San Jose, CA). The percentage of migration in each sample (% input) was determined from numbers of the starting cells and the migrated cells by the equation: [(No cells^Sample^/No cells^Input^) × 5] × 100.

### Tumor establishment in C57BL/6J and nude mice

Hepal-6 cells (2 × 10^6^ cells/200 μl), infected by rAAV-SLC or rAAV-GFP virus or noninfected as mock control were injected subcutaneously (s.c.) into the right flank of 6–8-week-old C57BL/6 J and nude mice. At the same time, intra-tumor (i.t.) injection of rAAV-SLC (5 μl, 0.6 **×** 10^9^ viral particles) into established tumors was studied. As previously indicated, the AAV viral vectors were delivered to six sites, 0.5 mm apart, along the needle track as the needle was withdrawn with a volume of ~0.8 μl at each of the six sites over 10 min. The needle was left in the tissue for an additional 5 min and then was slowly withdrawn. Developing tumors were monitored every day for 45 days. Tumors were harnessed on the day of 20 and weighed.

### Western blot analysis for SLC expression in the tumor sites

The harnessed tumor tissues were lysed with lysis buffer (RIPA) on ice. After centrifugation at 14,000 rpm at 4 °C for 30 min, supernatants were collected and total protein concentrations were determined by BCA assay (Pierce, Rockford, IL). Equal amounts of denatured proteins were loaded onto 10 % SDS-PAGE gel and transferred on PVDF membrane (Millipore, Bedford, MA). Membranes were blocked with 5 % nonfat milk in TBST (1× TBS containing 0.1 % Tween 20), then incubated with primary antibodies to SLC and β-actin (eBioScience, San Diego, CA) overnight, After three times washing with TBST, HRP-conjugated secondary antibodies (KPL, Gaithersburg, MD) were bound and performed with chemiluminescence using SuperSignal West Pico substrate (Pierce, Rockford, IL).

### IHC and flow cytometry analysis of tumor-infiltrating T cells and CD11c+ DCs

For analysis of cells infiltration, some tumors were harvested, removed of extraneous tissues on the day 20. Half of each tumor sample was used for OCT embedding and Serial 5 μm-thick Cryostat sections were performed by incubation with mAbs (PharMingen, San Diego, CA, USA) recognizing CD11c+ dendritic cells, CD4+Tcells, CD8+T cells as well as Foxp3+Tregs. Isotype-matched antibodies were used as a control. The remaining half was digested for 2 h at room temperature in 1 mg/ml type IV collagenase (Sigma, USA) with constant stirring. Digested tumors were passed through a 70 μm nylon mesh, washed once with HBSS, and resuspended in PBS/3 % BSA to ~1 × 10^6^ cells/ml. Polystyrene beads (15-μm diameter) were added to the samples to achieve a concentration of 5 × 10^5^ beads/ml. Samples were stained for the presence of FITC-conjugated CD25, PE-conjugated antibodies CD4, CD8, CD11c and Foxp3. Samples were analyzed by flow cytometry analysis with counting of 50,000 lymphocyte-sized events (based on splenocyte controls). The number of infiltrating CD4+ or CD8+cells was determined by the following equation: (No of PE-events/No of bead events) × 5 × 510^5 ^× 5 cell sample column.

### Statistical analysis

Statistical analyses were performed with software from SPSS 10.0 for Windows, Inc. (Chicago, IL). Results were evaluated by Student’s *t* tests. A *P* value  < 0.05 was taken as the level of significance.

## Results

### High transduction efficiency of liver cancer cells using rAAV-SLC

**Fig. 1:**
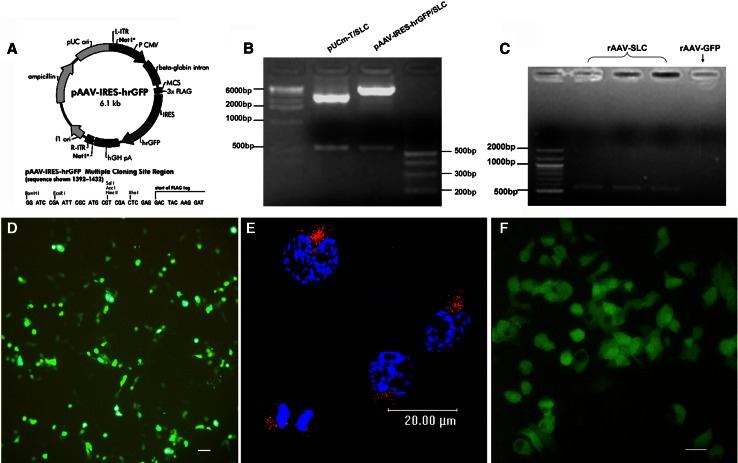
rAAV-SLC was constructed and infected Hepal-6 cells at high efficiency. **a** The entire nucleotide sequence of SLC was cloned into the MCS of the pAAV-IRES-hrGFP vector; Positive clones were demonstrated by restriction digestion (**b**) and by PCR (**c**); (**d**) rAAV-SLC was packaged in AAV293 cells by using the three-plasmid co-transfection system. GFP expression was served as a useful expression marker; **e** distribution of Cy3-rAAV-SLC particles (*red*) in Hepal-6 cells 2 h post-transduction were observed to shift toward the nucleus and begin to accumulate at the nuclear envelope, detected by laser scanning confocal microscope. The position of the cell nucleus was assessed by DAPI (*blue*) staining. A representative image is shown, consisting of a single plane of focus through the center of a cell; **f** 72 h post-transduction, SLC expression in Hepal-6 cells were analyzed by fluorescence microscopy for GFP detection

AAV Help-Free System was used for the package of rAAV-SLC. Firstly, the entire nucleotide sequence of SLC was cloned into the MCS of the pAAV-IRES-hrGFP vector. Positive clones was demonstrated by restriction digestion and PCR, as shown in Fig. 1a–c. As followed, a new rAAV 2 encoding SLC peptide (rAAV-SLC) was successfully packaged in AAV293 cells by three-plasmids co-transfection system, as shown in Fig. 1d, 72 h post-transfection, the virus particles was harvested. Virus titer was 1.25 × 10^11^ analysed by Real-time PCR, which was used to infect Hepal-6 cells later, with GFP as a marker of gene expression. Hepal-6 cells are highly susceptible to rAAV-mediated transduction. 2 h post-transfection the viral particles were observed to shift toward the nucleus and begin to accumulate at the nuclear envelope (Fig. 1e). 72 h post-transfection, based on the expression the marker gene GFP, it was found that about 50 % Hepal-6 tumor cells were positive in showing the transduction efficiency (Fig. 1f). There was no difference in transduction efficiency between the rAAV-SLC and rAAV-GFP. The control groups of single rAAV transduction have black background. The expression of SLC and its bioactivities were demonstrated in intro by ELISA and chemotaxis assay (Data not shown).

### rAAV-SLC induced a potent anti-tumor effect

**Fig. 2:**
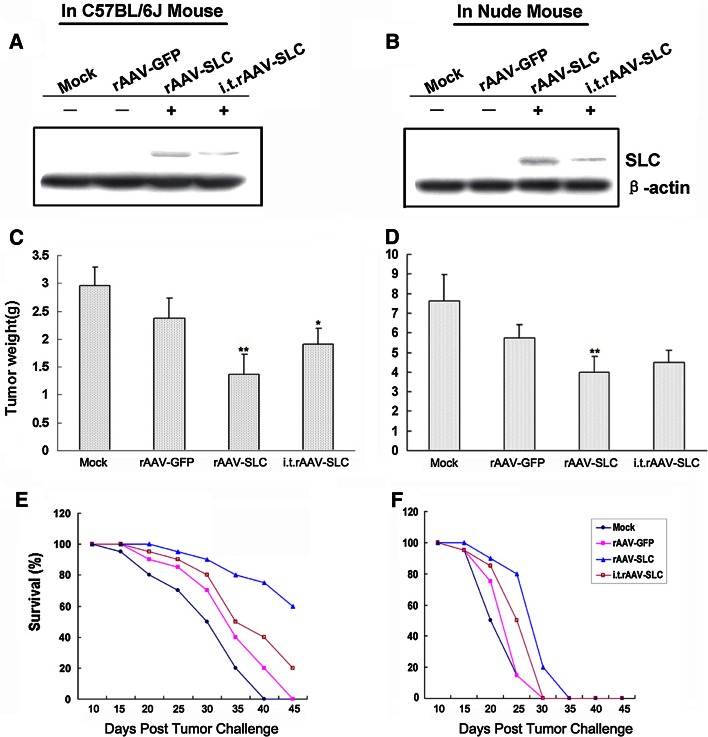
rAAV induced potent antitumor effect in vivo. **a** In C57BL/6J mice and **b** nude mice, the local expression of SLC in tumor sites was analyzed by Western-blot assay; **c**, **d** SLC-producing tumors showed a delayed progression and restrained in tumor weight when compared to rAAV-GFP tumors. No difference was observed between GFP-expressing and mock background tumors; **e**, **f** SLC-production was also associated with significantly improved survival. There was a worse survival in nude mice models, compared with C57BL/6J mice models. *Bars* SD, **p < 0.01 compared with rAAV-GFP group, *p < 0.05 compared with rAAV-SLC group; i.t., intra-tumor injection

The local expression of SLC in tumor sites of tumor bearing mice models were analyzed by Western-blot assay, we found the local expression of SLC in the tumors established from Hepal-6 cell lines previously transfected by rAAV-SLC. There was lower level local expression of SLC in the group of intra-tumor (i.t.) injection of rAAV-SLC into established tumors, as shown in Fig. 2a, b. The effect of rAAV-induced local expression SLC to enhance antitumor immune responses was subsequently tested. To elucidate in detail the mechanism of rAAV-SLC anti-tumor effect, we also examined its effect in nude mice. SLC-producing tumors showed an obviously delayed progression when compared to rAAV-GFP tumors (*p* < 0.01). rAAV-SLC had stronger anti-tumor effect compared with i.t.rAAV-SLC treated group (*p* < 0.05) in C57BL/6J mice. No difference was observed between GFP-expressing and normal background tumors, as shown in Fig. 2c, d. In parallel to reduced growth, SLC local expression was also associated with significantly improved survival as shown in Fig. 2e, f. The development of tumors was faster in nude mice than in C57BL/6J mice, and showed poorer survival.

### Local expression of SLC promoted the recruitment of T cells and DCs to the tumors in vivo

**Fig. 3:**
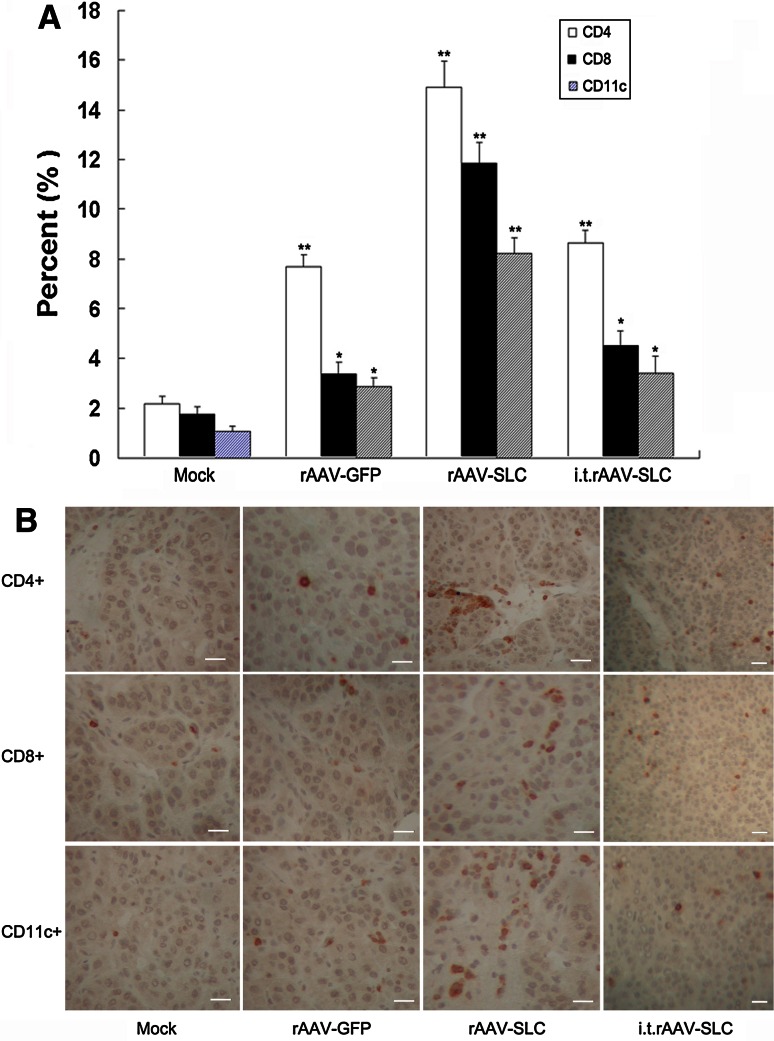
The observed antitumor effect of rAAV-SLC was immune-mediated. **a** The collected cells from tumor samples were stained FITC-CD11c, FITC-CD3 together with PE-CD4 or PE-CD8 for flow cytometric evaluation. Compared to control and rAAV-GFP, significantly higher numbers of CD11c+dendritic cells, CD4+T cells and CD8+T cells infiltration in SLC-treated mice were observed. *Bars* SD, *p < 0.05, **p < 0.01; i.t., intra-tumor injection; **b** serial 5 μm-thick Cryostat sections of tumor samples were performed by incubation with mAbs recognizing CD11c+dendritic cells, CD4+T cells and CD8+T cells. Positive cells were detected by DAB staining

Because SLC is chemotactic for T cells and DCs, we hypothesized that SLC expressed from tumor cells would elicit migration of these cells to the tumor site. To quantify the number of infiltrating CD4+ and CD8+T cell lymphocytes as well as CD11c+DCs were determined by flow cytometry and IHC analysis. These analysis illustrated that SLC-expression induced significantly higher numbers of CD4+, CD8+T cells and CD11c+DCs recruitment when compared to control (*p* < 0.01, as shown in Fig. 3). A modest increase in infiltrated immune cells was also observed in rAAV-GFP pre-infected tumors.

### Local expression of SLC also promoted higher infiltration of CD25+Foxp3+regulatory T cells (Tregs) in the tumor sites

**Fig. 4:**
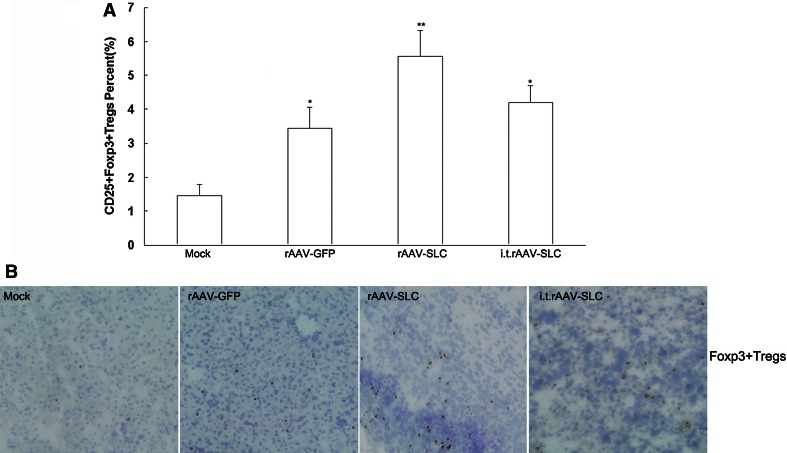
More Foxp3+regulatory T cells (Tregs) in the tumor sites were detected by FACS and IHC. **a** The collected cells from tumor samples were stained with FITC-CD25, PE-Foxp3 for flow cytometric evaluation of Tregs. Compared to control and rAAV-GFP, significantly higher numbers of Treg cells infiltration in mice with rAAV-SLC treatment. *Bars* SD, *p < 0.05, **p < 0.01; i.t., intra-tumor injection. **b** For IHC analysis of regulatory T cells infiltration, tumors cryostat sections were performed by incubation with mAbs recognizing Foxp3. Positive cells were detected by DAB. More Foxp3 positive cells were found in the tumor sites of mice with rAAV-SLC treatment

In order to further explore the mechanism of SLC mediated anti-tumor effect, infiltrated CD25+Foxp3+Tregs in the tumor sites were also tested by FACS (Fig. 4a) and IHC (Fig. 4b). There were more infiltrated Tregs in the groups of SLC local expression (*p* < 0.01).

## Discussion

In several studies, cytokine genes have been introduced into tumor cells in an attempt to provide a microenvironment that favors innate and acquired immune mechanisms to prevent or reverse tumor development [28]. In particular, chemokine gene transfer offers the possibility to trigger the recruitment of initiators or effectors of the immune response within the tumor [29, 30]. The chemokine m6Ckine/SLC has been proposed to play a role in favoring the interactions between dendritic cells and T cells in secondary lymphoid organs, through its interaction with the CCR7 receptor [9, 12, 16, 31–33]. Previous works demonstrated that SLC chemotactic activity for DCs and T cells could be harnessed to generate antitumor immune responses [20, 34–38]. Almost all of the past reports showed that antitumor effect of SLC was mediated by greatly enhancing the tumor infiltration of mature dendritic cells and CD8+T cells [39–41].

We reasoned that such an activity would be additionally improved by in situ activation of recruited DCs and T cells via local expression of SLC. In this study, we applied rAAV vector to deliver SLC directly into the tumor cells for its broad host range, excellent safety profile, and the ability to integrate into the host genome as well as long-term expression in infected hosts [42, 43]. Different from other reports [21, 34, 38, 44], we harnessed long-term local elaboration of SLC in the tumor bed mediated by rAAV vector to detect its antitumor effect. We have shown that rAAV-SLC was more effective in generating systemic antitumor responses and was accompanied by extensive CD4+, CD8+T cells, as well as CD11c+DCs infiltrating into the tumor sites. This is consistent with previous studies. On the other hand, the results also suggest that SLC local expression in tumor bed plays another role in anti-tumor effect on destroying the solid tumor barrier by infiltrating extensive lymphocytes. The ‘barrier’ formed around solid tumors by infiltrating host stroma hinders the presentation of tumor antigens in DLNs and, in the absence of a robust co-stimulatory environment, specific T cells can remain ignorant [45, 46]. Additional obstacles for T cell homing and expansion inside tumors may also prevent tumor eradication [47]. Murine models have shown that abundant, activated, tumor antigen–specific T cells fail to reject tumors even when they can reject skin grafts or less established tumors bearing the same antigen [48]. Thus, the tumor barrier may be an important contributor to the failure of tumor rejection. It is possible that the active recruitment of naïve T cells into tumors, followed by their activation through robust co-stimulation and sufficient antigen load, may overcome these obstacles in antitumor immunity.

However, we also found there was higher infiltration of Foxp3+ regulatory T cells (Tregs) mediated by rAAV-SLC treatment. It has been thoroughly demonstrated that during the tumor development, tumor microenvironment (such as the secretion of TGF-β and IL-10) can educate T cells into regulatory T cells (Treg) or more and more T cells became ignorant. Tregs are identified as a small proportion of CD4+T cells that highly express CD25 (IL-2R α-chain) on their surface and specifically express Foxp3 [49–53]. These CD4+CD25+Foxp3+Tregs act in a regulatory way by suppressing the activation of other T cells and control the immune responses induced by DCs in vivo [54, 55]. It has especially been shown Tregs prevent CD8+T cell maturation by inhibiting CD4+T cells in tumor sites [56]. Therefore, the infiltration of CD4+CD25+Foxp3+Tregs is a major obstacle for developing effective antitumor treatments. So in this study, the observed higher infiltration of Tregs in tumor sites played an important role in the tampered anti-tumor effect of SLC treatment. Further study is followed.

In summary, our study indicates that mouse SLC gene transferred into liver cancer cell line by rAAV has strong T cell mediated anti-tumor effects. Our work also suggests a comprehensive immune therapy strategy applicable for solid tumors based on cooperative interaction of multiple effector cell types including Tregs. This strategy may overcome functional immune impairment observed in patients with advanced malignancy.
